# TiO(OH)_2_ – highly effective catalysts for optimizing CO_2_ desorption kinetics reducing CO_2_ capture cost: A new pathway

**DOI:** 10.1038/s41598-017-03125-w

**Published:** 2017-06-07

**Authors:** Hongbao Yao, Sam Toan, Liang Huang, Maohong Fan, Yujun Wang, Armistead G. Russell, Guangsheng Luo, Weiyang Fei

**Affiliations:** 1 Department of Chemical and Petroleum Engineering, University of Wyoming, Laramie, WY 82071 USA; 2 State Key Laboratory of Chemical Engineering, Department of Chemical Engineering, Tsinghua University, Beijing, 100084 China; 3 State Key Laboratory of Refractories and Metallurgy, Wuhan University of Science and Technology, Wuhan, 430081 China; 4 School of Civil and Environmental Engineering, Georgia Institute of Technology, Atlanta, Georgia 30332 USA

## Abstract

The objective is to find a new pathway for significant reduction in CO_2_ capture energy consumption. Specifically, nanoporous TiO(OH)_2_ was used to realize the objective, which was desired as a catalyst to significantly accelerate the decomposition of aqueous NaHCO_3_, essentially CO_2_ desorption – the key step of Na_2_CO_3_/NaHCO_3_ based CO_2_ capture technologies from overall CO_2_ energy consumption perspective. Effects of several important factors on TiO(OH)_2_-catalyzed NaHCO_3_ decomposition were investigated. The quantity of CO_2_ generated from 0.238 mol/L NaHCO_3_ at 65 °C with catalyst is ~800% of that generated without the presence of catalyst. When a 12 W vacuum pump was used for carrying the generated CO_2_ out of reactor, the total amount of CO_2_ released was improved by ~2,500% under the given experimental conditions. No significant decrease in the catalytic effect of TiO(OH)_2_ was observed after five cyclic CO_2_ activated tests. In addition, characterizations with *in-situ* Fourier transform infrared spectroscopy, thermal gravity analysis and Brunauer-Emmett-Teller of TiO(OH)_2_ indicate that TiO(OH)_2_ is quite stable. The discovery in this research could inspire scientists’ interests in starting to focus on a new pathway instead of making huge effort or investment in designing high-capacity but expensive CO_2_ sorbent for developing practical or cost-effective CO_2_ technologies.

## Introduction

There is no doubt that countless progresses have been made in controlling SOx/NOx and Hg emissions from fossil fuel fired power plants^1^. However, the actions on CO_2_ emission control have been slow^2^. The importance of CO_2_ capture in fossil fuel-fired power plants cannot be underestimated any more due to the catastrophic effect of the continuous increase in CO_2_ concentration in atmosphere and the ill effects it has on the environment^3–5^, as indicated in the recently reached Paris Agreement^6^.

Nowadays, chemical absorption by liquid solvents, including amines and carbonates, is widely considered to be the most promising method among various post-combustion CO_2_ capture technologies^7–9^. However, high-energy requirements in the regeneration or CO_2_ desorption process is the largest obstacle for preventing its industrial applications^10^. Conventional regenerations of spent solvents are just realized by heating spent solvents above boiling temperatures (100–150 °C). The energy consumption needed for CO_2_ desorption or spent sorbent regeneration accounts for ~15–30% of power plants’ electricity outputs^11^.

Accordingly, many other efforts have been made for lowering CO_2_ desorption energy consumptions and up to date, there mainly exists three important strategies^10–12^. The most popular one is to use new organic amine mixtures for CO_2_ sorption and desorption. It was generally believed that adsorption solvents with lower heat of absorption require less heat to be regenerated. Thus, much attention has been focused on the blending of different types of amine solutions^12–14^. The other two strategies include the application of novel equipment with superior mass and heat transfer performance as well as process optimizations^12, 15^. For example, stripping CO_2_ from aqueous potassium carbonate solutions was achieved by using two types of polymeric flat sheet microporous membrane contactors, which have been reported by Michael and co-workers^16^. Unfortunately, the efforts made in promoting CO_2_ desorption or spent sorbent regeneration are still much less than that improving CO_2_ absorption.

On the other hand, it should be pointed out that amine solvents, such as monoethanolamine (MEA), diethanolamine (DEA) and methyldiethanolamine (MDEA), have several serious shortcomings such as toxicity, corrosiveness, and oxidative degradation^17, 18^. Alternative sorbents are inorganic carbonate solutions based on Equation R1, in which M stands for Na or K.

R1 $${{\rm{M}}}_{2}{{\rm{CO}}}_{3}+{{\rm{CO}}}_{2}+{{\rm{H}}}_{2}{\rm{O}}\leftrightarrow 2{{\rm{MHCO}}}_{3}.\,$$

R1 based CO_2_ captures are not only inexpensive but also stable and thus environmentally friendly. However, like any other CO_2_ sorbents, its slow CO_2_ desorption kinetics make its wide utilization unaffordable, considering the fact that the energy consumption of the CO_2_ desorption step accounts for ~70–80% of those needed for the corresponding overall CO_2_ capture processes^19^.

This research was designed to fill the gap with its focus on investigating the significant effect of TiO(OH)_2_ on promoting aqueous NaHCO_3_ decomposition or CO_2_ desorption kinetics and the cost of Na_2_CO_3_/NaHCO_3_ based CO_2_ capture technology. The success could lead to a new pathway for future CO_2_ capture technology development with its focus on catalysis instead of high-capacity but expensive sorbents.

## Results

### Effects of several key factors on the performance of TiO(OH)_2_ on NaHCO_3_ decomposition, essentially CO_2_ desorption

#### Stirring rate

**Figure 1:**
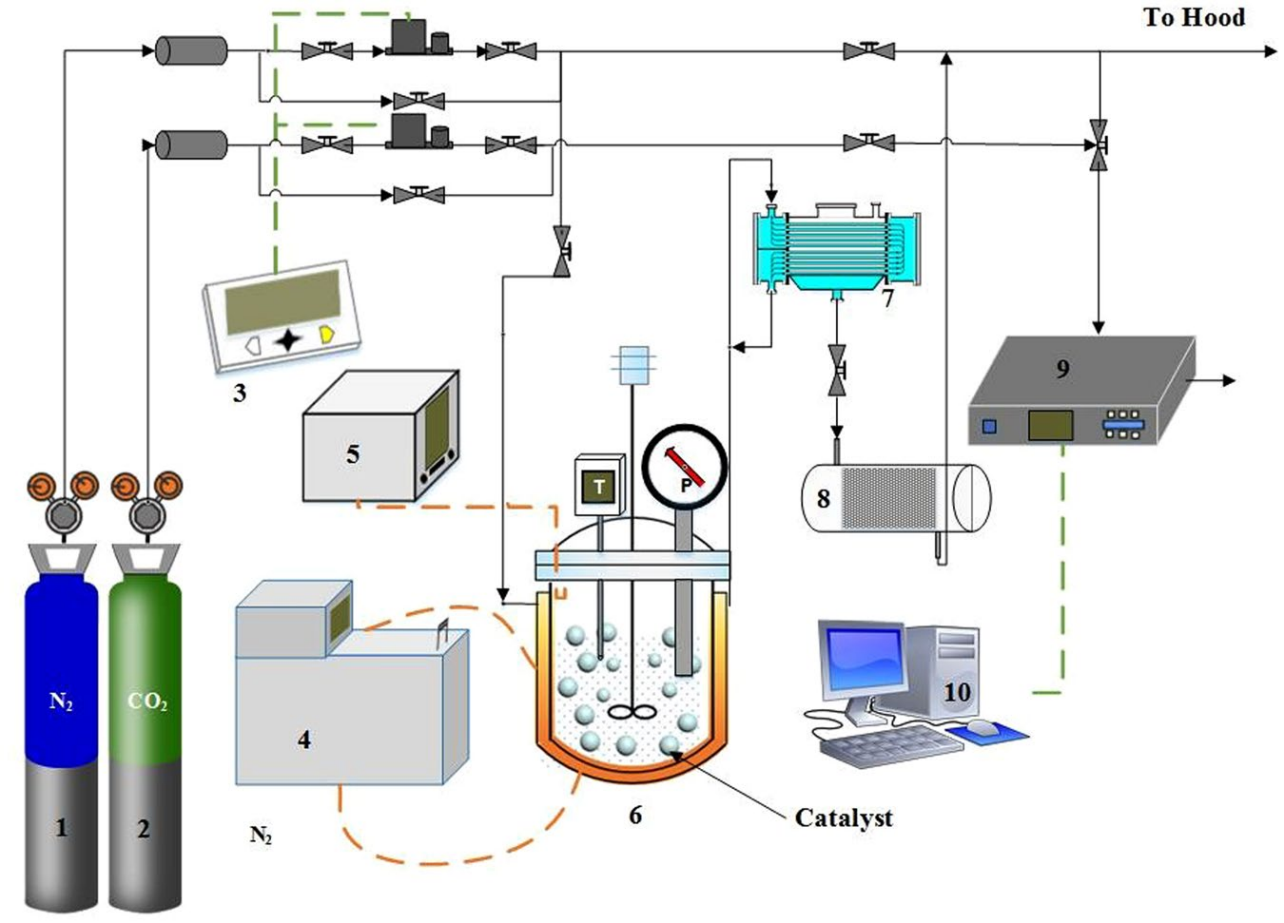
Schematic diagram of CO_2_ desorption set-up [1: N_2_ cylinder; 2: CO_2_ cylinder; 3: mass flow controller; 4: thermostatic water bath; 5: thermoelectric couple; 6: stirred tank reactor; 7; condenser; 8: desiccator; 9; gas concentration analyzer; 10: data recording unit].

**Figure 2:**
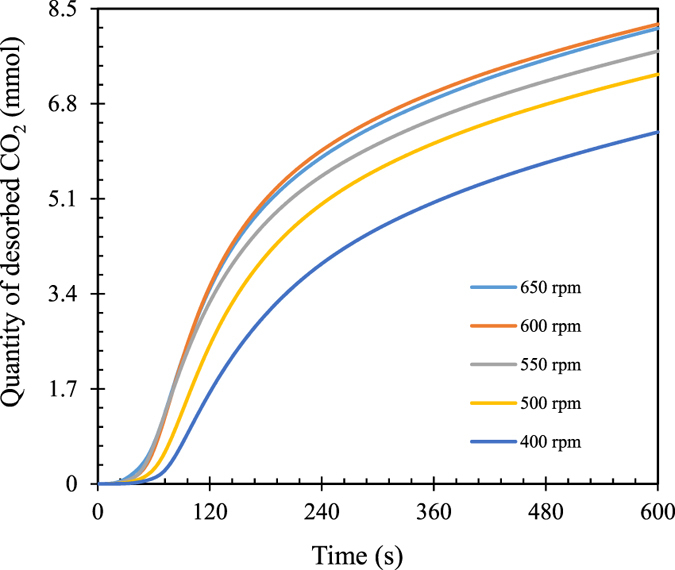
NaHCO_3_ decomposition or CO_2_ desorption profiles under different stirring rates [initial NaHCO_3_ concentration: 0.143 mol/L; Ti/Na molar ratio: 0.78; reaction temperature: 70 °C; N_2_ flow rate: 500 mL/min].

The experimental set-up for aqueous NaHCO_3_ decomposition or CO_2_ desorption test is illustrated in Fig. 1. The photo of actual experimental set-up is provided in supporting information (SI Photo 1). The quantities of CO_2_ desorbed under different stirring rates at the given temperature (70 °C) were first evaluated. As shown in Fig. 2, CO_2_ desorption amounts increase with the increase in stirring rates ranging from 400 rpm to 550 rpm while stirring rate only shows slight effect when it increases from 600 rpm to 650 rpm. This means that the mass transfer resistance of CO_2_ diffusing from liquid phase to gas phase was intensively reduced and thus, CO_2_ desorption is dominated by its reaction kinetics when stirring rate is higher than 600 rpm under experimental conditions.

#### Quantity of TiO(OH)_2_ added

**Figure 3:**
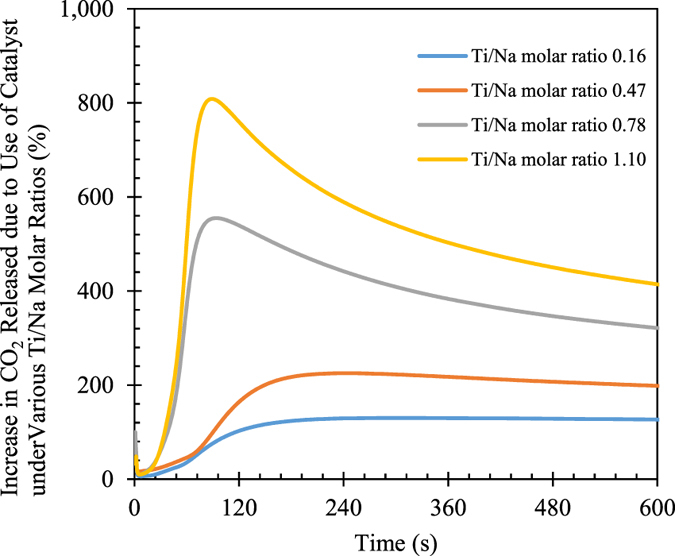
Effect of Ti/Na molar ratio on NaHCO_3_ decomposition or CO_2_ desorption [initial NaHCO_3_ concentration: 0.238 mol/L; stirring rate: 600 rpm; reaction temperature: 65 °C; N_2_ flow rate: 500 mL/min].

The effect of the quantity of TiO(OH)_2_ or the Ti/Na molar ratio in solution on the decomposition of NaHCO_3_ is shown in Fig. 3. NaHCO_3_ decomposition or CO_2_ desorption significantly increases with the quantity of added TiO(OH)_2_ or the Ti/Na ratio. However, the improvement sensitivity decreases with Ti/Na ratio. Under the test conditions, the highest improvement in NaHCO_3_ decomposition or CO_2_ desorption reaches 800% at 110 s of reaction time. Considering the fact that the quantity of H_2_O in reactor is much more than that stoichiometrically needed, the promotional effect of TiO(OH)_2_ on NaHCO_3_ decomposition or CO_2_ desorption is exceptional.

#### Temperature

**Figure 4:**
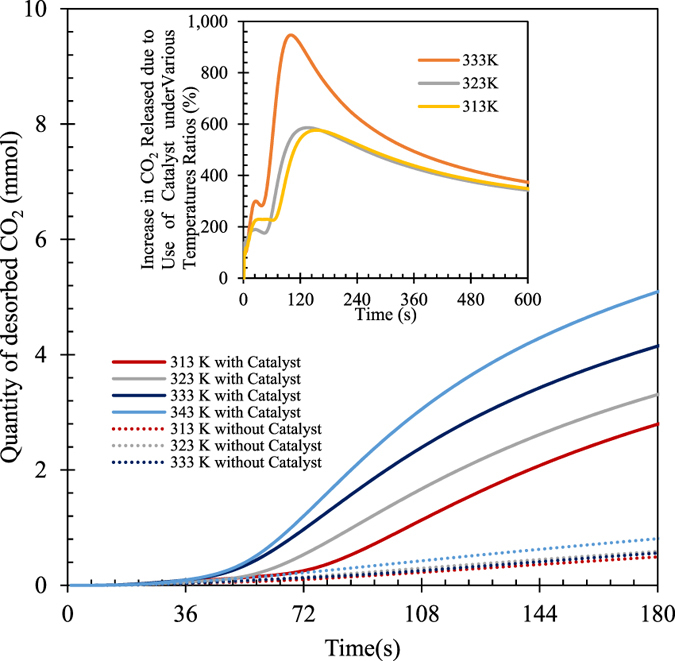
Effect of temperature on catalytic NaHCO_3_ decomposition or CO_2_ desorption [initial NaHCO_3_ concentration: 0.143 mol/L; Ti/Na molar ratio: 0.78; stirring rate: 600 rpm; N_2_ flow rate: 500 mL/min].

The effects of TiO(OH)_2_ on NaHCO_3_ decomposition or CO_2_ desorption kinetics at temperatures of 40 °C, 50 °C, 60 °C and 70 °C are presented in Fig. 4. The quantity of CO_2_ released within the first 180 seconds with the use of TiO(OH)_2_ at 40 °C is 2.79 mmol, ~510% higher than that obtained without use of TiO(OH)_2_ under the same condition. At 70 °C, the catalyst can improve NaHCO_3_ decomposition by about 490% within the first 180 s. The catalytic effects gradually decrease with time as dictated with thermodynamic theories, although they are still considerably large after 600 s of NaHCO_3_ decomposition. For example, 5.63 mmol of CO_2_ was released with the use of TiO(OH)_2_ at 40 °C after 600 s, while only 1.61 mmol of CO_2_ was released without presence of TiO(OH)_2_under the same conditions, a 71.4% decrease.

### Cyclic performance of TiO(OH)_2_ on NaHCO_3_ decomposition

**Figure 5:**
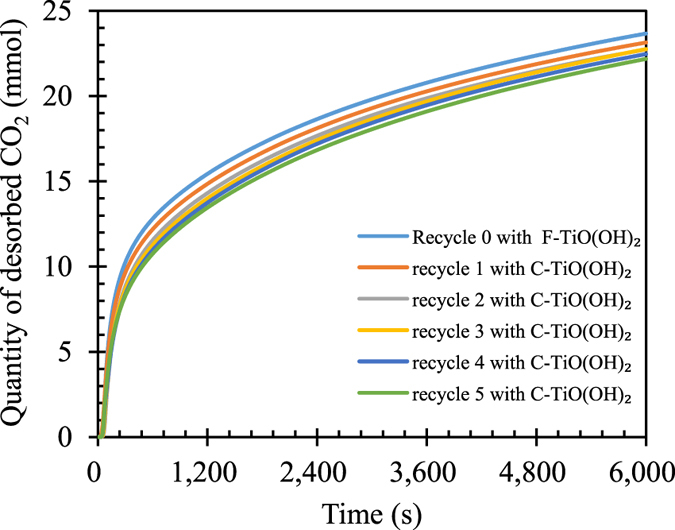
Cyclic performance of TiO(OH)_2_ on NaHCO_3_ decomposition [initial NaHCO_3_ concentration: 0.238 mol/L; Ti/Na molar ratio: 0.78, stirring rate: 600 rpm; reaction temperature: 60 °C, N_2_ flow rate: 500 mL/min].

In addition, the regeneration performance of NaHCO_3_ solution decomposition using TiO(OH)_2_ plays a key role in practical applications and cannot be neglected, which has also been examined in detail in this work. The fresh TiO(OH)_2_ and CO_2_-treated TiO(OH)_2_ or cycled TiO(OH)_2_ are hereafter denoted as F-TiO(OH)_2_ and C-TiO(OH)_2_, respectively. The C-TiO(OH)_2_ promoted CO_2_ release curves obtained from aqueous NaHCO_3_ are almost overlap and no remarkable decrease in the total CO_2_ desorption amounts are noticed in five continuous cycles as shown in Fig. 5. Furthermore, considering the resulting pore structure data summarized in Table 1, the specific surface area and pore volume of C-TiO(OH)_2_ after 5 cycles of uses are 693.1 m^2^/g and 0.658 cm^3^/g, respectively, are comparable to those of F-TiO(OH)_2_. This suggests that TiO(OH)_2_ is a stable catalyst for NaHCO_3_ decomposition or CO_2_ desorption.

**Table 1:** BET characteristics of F-TiO(OH)_2_ and C-TiO(OH)_2_.

	Specific surface area (m^2^/g)	Pore volume (cm^3^/g)	Pore diameter (Å)
F-TiO(OH)_2_	807.4	0.73	17.12
C-TiO(OH)_2_ after 5 cycles	693.1	0.66	17.08

## Discussion

N_2_ adsorption/desorption isotherms and pore-size distributions were conducted to investigate the pore structures of F-TiO(OH)_2_ and C-TiO(OH)_2_ with 5 cycles of CO_2_ desorption and sorption. The resulting pore structure data were summarized in Table 1. Both samples are nanoporous with the average diameter close to 1.7 nm. The specific surface area of F-TiO(OH)_2_ could reach to as high as 807.4 m^2^/g, while that of C-TiO(OH)_2_ is 693.1 m^2^/g, about a 14% drop.

**Figure 6:**
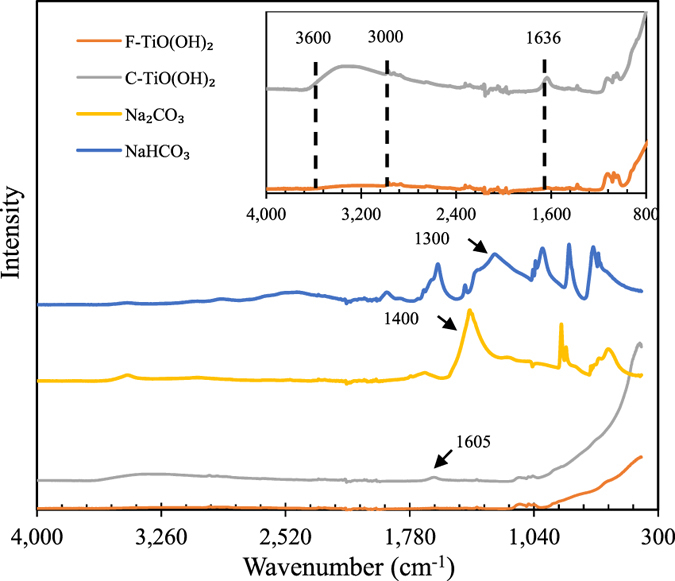
FT-IR spectra of F-TiO(OH)_2_, C-TiO(OH)_2_, Na_2_CO_3_, and NaHCO_3_.

The FT-IR absorption spectra of two TiO(OH)_2_ samples, Na_2_CO_3_ and NaHCO_3_ are shown in Fig. 6. Both TiO(OH)_2_ samples show broad peaks in the 400–900 cm^−1^ range corresponding to Ti-O bending. The additional peaks in the 3,000–3,600 cm^−1^ range existing in C-TiO(OH)_2_ sample are likely due to the partial hydration during the reaction process. Typically, two small peaks at 1,124 and 1,072 cm^−1^ in the F-TiO(OH)_2_ likely result from Ti-O-C, the ending and bridging isopropyl groups, considering that TiO(OH)_2_ are directly synthesized from the hydrolysis of TTIP. Similar phenomena were also reported by Sui and coworkers^20^. Small peaks at 1,132, 1,117 and 1,022 cm^−1^ were associated with Ti-O-C and butoxyl groups. In addition, it should be noted that C-O-H bending vibrations could appear as a broad and weak peak at 1,440–1,220 cm^−1^ in alcohols and phenols^21, 22^. Accordingly, the two small peaks observed at 1,124, 1,072 cm^−1^ for F-TiO(OH)_2_ and C-TiO(OH)_2_ may also be related to Ti-O-H group, which may be the major player in accelerating NaHCO_3_ decomposition or CO_2_ desorption.

Moreover, the FT-IR spectra of the NaCO_3_ and NaHCO_3_ obtained in experiments are consistent with those in references^23–25^. The noteworthy peaks at 1,300 cm^−1^ and 1,400 cm^−1^ for NaHCO_3_ and Na_2_CO_3_, respectively, are due to carbonate asymmetric stretching^26^. In addition, a peak at 1,605 cm^−1^ was observed for NaHCO_3_, which can be attributed to symmetric stretching of CO_2_. Therefore, the peak at 1,636 cm^−1^ for C-TiO(OH)_2_ may be due to the presence of CO_3_^2−^ or HCO_3_^−^. This indicates that NaHCO_3_ or NaCO_3_ may be sorbed on the inner pore of F-TiO(OH)_2_, which could be responsible for the decrease in specific surface area of C-TiO(OH)_2_ as observed in Table 1.

**Figure 7:**
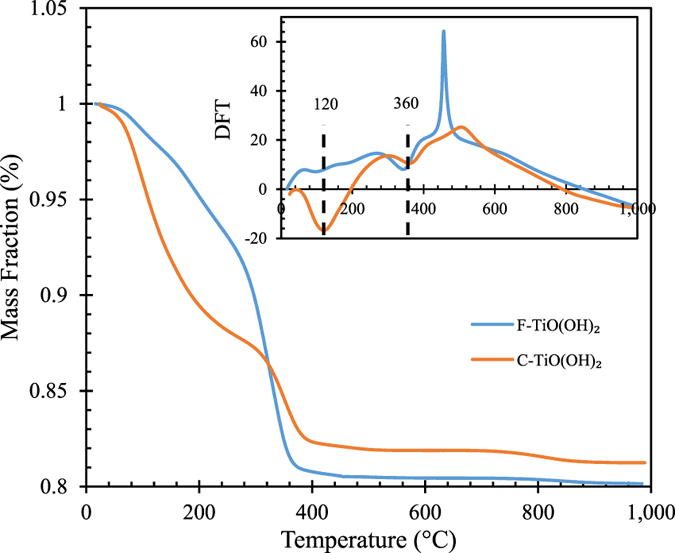
TGA data of F-TiO(OH)_2_ and C-TiO(OH)_2_ [heating rate: 20 °C/min; N_2_ flow rate: 100 mL/min].

The TGA data of F-TiO(OH)_2_ and C-TiO(OH)_2_ are shown in Fig. 7. Both samples started to lose absorbed water at ~120 °C, and then decomposed to TiO_2_ above 300 °C. The final masses of F-TiO(OH)_2_ and C-TiO(OH)_2_ account for 80.1% and 81.2% of their initial masses, respectively, indicating that no titanium loss took place during the NaHCO_3_ decomposition or CO_2_ desorption process in this research. F-TiO(OH)_2_ shows slower mass loss rates at lower temperatures, which could be due to loss of organic groups^27^ from TTIP. This agrees with what was observed in the FTIR results.

**Figure 8:**
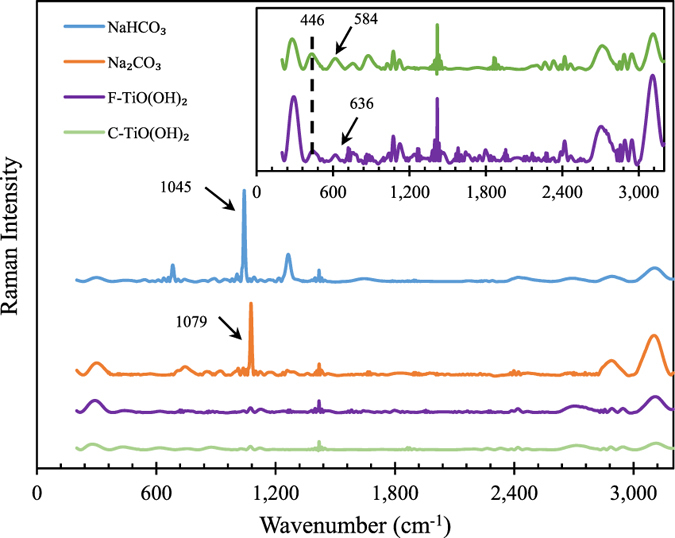
Raman spectra of F-TiO(OH)_2_, C-TiO(OH)_2_, pure Na_2_CO_3_ and NaHCO_3_.

Furthermore, Raman spectroscopy analyses of F-TiO(OH)_2_, C-TiO(OH)_2_, pure Na_2_CO_3_ and NaHCO_3_ were also conducted and the results are shown in Fig. 8. All samples have a similar broad band in the range of 3,000–3,200 cm^−1^, which resulted from H-O vibration in the water absorbed from air^28^. Two very strong peaks at *ca* 1,043 cm^−1^ for NaHCO_3_ and at *ca* 1,079 cm^−1^ for Na_2_CO_3_ were observed, which has been reported by others^29, 30^ and can both be assigned to the *ν*_*1*_ symmetric stretch.

Both TiO(OH)_2_ samples show relatively intense Raman bands at *ca* 446 cm^−1^ and *ca* 636 cm^−1^ (*ca* 584 cm^−1^ for reacted TiO(OH)_2_). Typically, the frequencies of the Raman bands observed are 513 cm^−1^ and 636 cm^−1^ for anatase and 446 cm^−1^ and 609 cm^−1^ for rutile, respectively^29, 31, 32^. The TiO(OH)_2_ sample in this work is amorphous and the position and intensity of its characteristic peaks can be changed by modifying its material structures. It should be pointed out that the resulting Raman spectrum for C-TiO(OH)_2_ is quite similar to that of F-TiO(OH)_2_, implying that TiO(OH)_2_ is stable or can be cyclically used.

**Figure 9:**
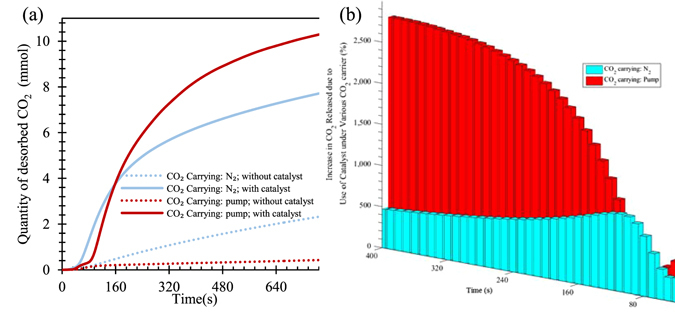
(**a**) Effect of different CO_2_ carrying out methods on CO_2_ release [initial NaHCO_3_ concentration: 0.238 mol/L; Ti/Na molar ratio: 0.78; stirring rate: 600 rpm; reaction temperature: 65 °C, N_2_ flow rate: 200 mL/min; power of pump: 12 W]. (**b**) Ratio of CO_2_ released by using N_2_ gas and the pump with and without uses of catalyst for each 10 seconds.

Finding a cost-effective method for carrying the released or desorbed CO_2_ out of reaction system is important. A pump was used to compare its performance on carrying the released CO_2_ out of its desorption system to that achieved with N_2_ as a carrier gas, and the results obtained within 55–65 °C are shown in Fig. 9. It can be seen that the quantity of CO_2_ carried out with N_2_ was high than that with a pump when NaHCO_3_ decomposition was not catalyzed with TiO(OH)_2_. However, the pump carried out 25% more CO_2_ than the same amount of N_2_ did when the catalyst was used. The quantity of the CO_2_ generated from non-catalytic NaHCO_3_ decomposition with 200 ml/min N_2_ being a carrier gas is only ~1/8 of that from catalytic NaHCO_3_. Furthermore, when a 12 W pump was used for carrying the generated CO_2_ out of NaHCO_3_ decomposition reactor, the CO_2_ releasing improvement due to the help of the catalyst is about 25 times or 2,500%.

**Figure 10:**
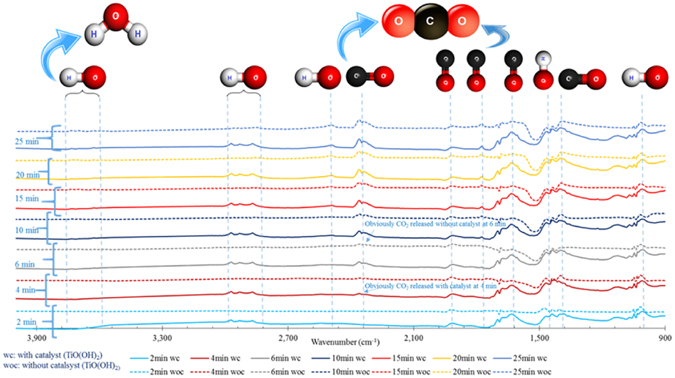
FT-IR- characterization of catalytic and non-catalytic solid NaHCO_3_ decomposition [NaHCO_3_: TiO(OH)_2_ mole ratio: 1:2; temperature: 150 °C; He flow rate: 5 ml/min].

Figures 3, 4 and 9 clearly show that noticeable CO_2_ release from catalytic NaHCO_3_ decomposition was observed earlier than that from non-catalytic NaHCO_3_ decomposition. This is not only confirmed with the experimental set-up shown in Fig. 1 but also with the data collected with the FTIR function of the available *in-situ* FTIR-MS (SI Photo 2) for solid NaHCO_3_ decomposition and shown in Fig. 10. The time needed for the appearing of the peak of C=O in the released CO_2_ of catalytic NaHCO_3_ decomposition is longer than that of non-catalytic NaHCO_3_ decomposition.

## Conclusion

Nanoporous TiO(OH)_2_ is a very effective catalyst for aqueous NaHCO_3_ decomposition or CO_2_ desorption, and thus CO_2_ capture. The finding could be significantly helpful for reducing the global concern about the high energy consumption required for CO_2_ emission control. Further works should focus on understanding the associated mechanism and extending the new concept to other CO_2_ emission and utilization technologies development.

## Methods

### TiO(OH)_2_ preparation

Titanium isopropoxide (TTIP), ethanol (EtOH) and sodium bicarbonate (NaHCO_3_) were purchased from Sigma-Aldrich and used without any further purification prior to preparing TiO(OH)_2_. The first TiO(OH)_2_ preparation step was to add 25 mL titanium isopropoxide to 350 mL deionized water at room temperature, followed by 4 hours of vigorous stirring. The resulting white precipitate was separated by filtration, and then washed several times with deionized water and anhydrous ethanol sequentially. The wet TiO(OH)_2_ was dried under 120 °C in an oven for 12 h.

### TiO(OH)_2_ characterization

The Brunauer-Emmett-Teller (BET) surface areas and pore size distribution of TiO(OH)_2_ samples were measured by using a Quantachrome Autosorb-iQ pore structure analyzer. Pore volumes were estimated from the adsorbed amount of N_2_ at a relative pressure of P/P_0_ = 0.99. Fourier transformed-infrared (FTIR) spectroscopy data were collected with a Thermo Nicolet Magna-IR 760 spectrometer (SI Photo 1). Thermal gravity analysis (TGA) tests were conducted on a TA Instruments SDT Q600 thermogravimetric analyzer. Raman spectra of the TiO(OH)_2_ samples were collected on a Raman Sierra IM-52 instrument from Snowy Range Instruments with a 532 nm laser and 3 mW power.

### NaHCO_3_ decomposition or CO_2_ desorption tests

NaHCO_3_ decomposition tests were started by adding predetermined amounts of TiO(OH)_2_, NaHCO_3_ and deionized water into the reactor stirred at the rate of 600 rpm. Reaction temperatures were regulated using a digital temperature controller. During NaHCO_3_ decomposition, pure N_2_ or vacuum pump (Karlsson Robotics, 12 W) was used to carry the desorbed CO_2_ out of reactor. The change of CO_2_ outlet concentration with time was recorded in a data acquisition unit, and the concentration-time profile was used to calculate the quantity of CO_2_ desorbed and evaluated to observe the difference in CO_2_ desorption due to the use of TiO(OH)_2_. When TiO(OH)_2_ was used for cyclic NaHCO_3_ decomposition or CO_2_ desorption tests, the used TiO(OH)_2_ and deionized water were mixed in a closed tank with pure CO_2_ at 200 psi followed by vigorous stirring under room temperature to study the effect of CO_2_ sorption or activation on TiO(OH)_2_.

## Electronic supplementary material

supplementary information
