# Supplementary material for: TiO(OH)2 – highly effective catalysts for optimizing CO2 desorption kinetics reducing CO2 capture cost: A new pathway
Source: Sci Rep. 2017 Jun 7;7:2943. doi: 10.1038/s41598-017-03125-w (PMC5462786; doi:10.1038/s41598-017-03125-w)
Supplement: Supplementary file 1 — supplementary information [file 41598_2017_3125_MOESM1_ESM.doc]

**TiO(OH)_2_ – highly effective catalysts for optimizing CO_2_ desorption kinetics reducing CO_2_ capture cost: A new pathway**

Hongbao Yao^a,b^, Sam Toan^a^, Liang Huang ^c^,Maohong Fan^a,d^*^1^, Yujun Wang^b^,*^2^, Armistead G. Russell^d^, Guangsheng Luo^b^, Weiyang Fei^b^

*^a^Department of Chemical and Petroleum Engineering, University of Wyoming, Laramie, WY 82071, USA*

*^b^State Key Laboratory of Chemical Engineering, Department of Chemical Engineering, Tsinghua University, Beijing 100084, China*

*^c^State Key Laboratory of Refractories and Metallurgy, Wuhan University of Science and Technology, Wuhan 430081, China*

*^d^School of Civil and Environmental Engineering, Georgia Institute of Technology, Atlanta, Georgia, 30332, USA.*

**Supporting Information**

**
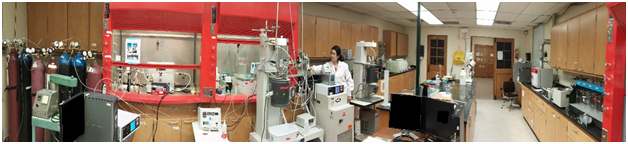
**

Photo 1 CO_2_ desorption or NaHCO_3_ decomposition experimental set-up


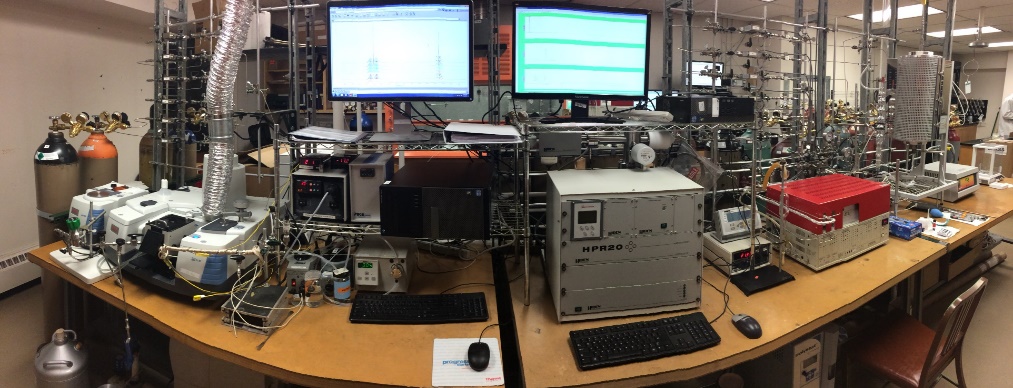
 Photo 2 Integrated FTIR-MS system used for studying CO_2_ release from NaHCO_3_
